# Interference Impacts Working Memory in Mild Cognitive Impairment

**DOI:** 10.3389/fnins.2016.00443

**Published:** 2016-10-13

**Authors:** Sara Aurtenetxe, Javier García-Pacios, David del Río, María E. López, José A. Pineda-Pardo, Alberto Marcos, Maria L. Delgado Losada, José M. López-Frutos, Fernando Maestú

**Affiliations:** ^1^Laboratory of Cognitive and Computational Neuroscience, Center for Biomedical Technology of Madrid (CBT), Universidad Complutense de Madrid and Universidad Politécnica de MadridMadrid, Spain; ^2^Department of Psychology, Faculty of Health Sciences, Camilo Jose Cela UniversityMadrid, Spain; ^3^Department of Basic Psychology II (Cognitive Processes), Universidad Complutense de MadridMadrid, Spain; ^4^Laboratory of Neuropsychology, Universitat de les Illes BalearsPalma de Mallorca, Spain; ^5^Centro Integral de Neurociencias AC, HM Puerta del Sur, Hospitales de Madrid MostolesMadrid, Spain; ^6^CEU San Pablo UniversityMadrid, Spain; ^7^Department of Neurology, San Carlos University HospitalMadrid, Spain; ^8^Seniors Centre of the District of ChamartínMadrid, Spain; ^9^Department of Basic Psychology, Universidad Autónoma de MadridMadrid, Spain

**Keywords:** mild cognitive impairment, working memory, interference, behavioral research, aging

## Abstract

Mild cognitive impairment (MCI) is considered a transitional stage between healthy aging and dementia, specifically Alzheimer's disease (AD). The most common cognitive impairment of MCI includes episodic memory loss and difficulties in working memory (WM). Interference can deplete WM, and an optimal WM performance requires an effective control of attentional resources between the memoranda and the incoming stimuli. Difficulties in handling interference lead to forgetting. However, the interplay between interference and WM in MCI is not well-understood and needs further investigation. The current study investigated the effect of interference during a WM task in 20 MCIs and 20 healthy elder volunteers. Participants performed a delayed match-to-sample paradigm which consisted in two interference conditions, distraction and interruption, and one control condition without any interference. Results evidenced a disproportionate impact of interference on the WM performance of MCIs, mainly in the presence of interruption. These findings demonstrate that interference, and more precisely interruption, is an important proxy for memory-related deficits in MCI. Thus, the current findings reveal novel evidence regarding the causes of WM forgetting in MCI patients, associated with difficulties in the mechanisms of attentional control.

## Introduction

Mild cognitive impairment (MCI) is considered a transitional stage between healthy aging and dementia, specifically Alzheimer's disease (AD). The most common clinical symptom of MCI is episodic memory loss, with a particularly rapid rate of forgetting and impaired delayed recall. However, deficits in working memory (WM) and executive functions are frequently observed in MCI populations as well (Albert et al., 2001; Huntley and Howard, 2010); specially in multi-domain subtype (Klekociuk and Summers, 2014). WM is affected by interference, and its optimal performance requires adequate executive mechanisms to control attention between the to-be-remembered stimuli and the interference (Sakai et al., 2002a,b). Difficulties in inhibitory control and attentional switching result in WM depletion in healthy aging (Clapp and Gazzaley, 2012). However, evidence on how interference my affect WM in MCI individuals is scarce and merits further investigation.

The inhibitory model proposed by Hasher and Zacks (1988) postulates that changes in the ability to ignore or control distracting information underlie cognitive deficits in aging. Indeed, increasing evidence correlates age-related memory decay with reduced ability to regulate interference (Zacks and Hasher, 1994; Hasher et al., 1999; Jonides et al., 2000; Hedden and Park, 2001; Darowski et al., 2008; Healey et al., 2008; Stevens et al., 2008). Two main categories of interference have been mostly explored in aging research: distraction and interruption (Solesio-Jofre et al., 2011; Clapp and Gazzaley, 2012). *Distraction* refers to irrelevant stimuli which need to be ignored (e.g., ignoring an alarm while calculating the expenses). *Interruption* refers to stimuli which demand additional processing as a secondary task (also considered multitasking, e.g., switching-off an alarm while calculating the expenses; Salvucci and Taatgen, 2008). Both stimuli share behavioral attributes because they both affect WM performance, but differ in the degree of impact (interruption has a greater impact on WM than distraction) and also in the underlying mechanisms that support them (Clapp and Gazzaley, 2012). Although both types of interference are handled by top-down process, distraction requires a controlled suppression/inhibition of the irrelevant stimulus, while interruption requires in addition a controlled attention switching mechanism. Based on this evidence, it becomes important to disentangle between these two categories of interference when studying the mechanisms responsible for WM depletion in MCI.

WM difficulties in MCIs have been observed through neuropsychological assessments (Saunders and Summers, 2010; see Huntley and Howard, 2010, for a neuropsychological review about early AD profiles) and experimental paradigms (Belleville et al., 2007, 2008a; Missonnier et al., 2007; Kochan et al., 2011). In addition, difficulties in inhibition, attention/task-switching and in resolving interference have been also evidenced through experimental procedures (Albert et al., 2001; Wylie et al., 2007; Belleville et al., 2008b; Bélanger and Belleville, 2009; Borkowska et al., 2009; Lonie et al., 2009; Sinai et al., 2010; Clément et al., 2012). In this line, several studies have evidenced that memory consolidation in MCI individuals is significantly affected by difficulties in memory control from interference (Della Sala et al., 2005; Dewar et al., 2009, 2012). For example, results from California verbal learning-like tests reflect the vulnerability to semantic interference in MCI patients, being a predictive factor of conversion to AD (Loewenstein et al., 2007; Rabin et al., 2009; Silva et al., 2012). Besides, the negative impact of interference has been also observed during short memory delay periods. Deiber et al. (2011) explored the neuronal response to distraction during WM in single- and multi-domain MCI patients. Results showed altered mechanisms for controlling distraction especially in the multi-domain subgroup of patients. In a similar manner, Alescio-Lautier et al. (2007) and Belleville et al. (2007) observed memory difficulties when dividing attention between a memory probe and an interfering stimulus in MCIs. Furthermore, those patients with more severe clinical status revealed greater vulnerability to interference. Based on the above mentioned studies, it becomes important to assess the impact of external stimuli when evaluating memory abilities in MCI populations. However, as far as we are aware of, there is no evidence showing how distraction and interruption differently affect WM performance within the same MCI individuals. Therefore, the present study aimed to explore the effect of two types of interference on WM in MCI and healthy elderly participants. We employed a visual delayed match-to-sample task with three conditions: non-interference, distraction and interruption. Under these circumstances, we expected: (a) reduced WM in all conditions in the MCI sample, and (b) higher impact of interruption than distraction on WM performance, especially in the MCI group.

## Methods

### Participants

A total of 40 volunteers were included in the study. All of the participants were over 65 years of age, right-handed (Oldfield, 1971) and native Spanish speakers. The participants were divided into two groups based on their clinical profiles: 20 MCI patients and 20 healthy elderly controls. The groups were matched for age; educational level and gender (see Table 1). MCI patients were recruited from the Hospital Universitario San Carlos, and control adult volunteers were recruited from the Seniors Center of the district of Chamartín, Madrid.

**Table 1:** **Demography and neuropsychology data (mean scores and standard deviation in parenthesis) are shown for controls (CNT) and MCI patients**.

	**N**	Left HV^*^	Right HV^*^	**Age**	**Sex**	MMSE^*^	**Education**	**GDS**	**FAQ**	**GDS-15**				
CNT	20	2.5 (0.4) × 10−3	2.5 (0.3) × 10−3	71.7 (2.8)	12F, 8M	29.4 (0.7)	3.7 (1.0)	1.0 (0.0)	0.05 (0.2)	2.2 (4.0)				
MCI	20	2.1 (0.4) × 10−3	2.1 (0.4) × 10−3	73.6 (3.5)	9F, 11M	28.3 (1.7)	3.0 (1.2)	3.0 (0.0)	0.8 (1.5)	2.0 (2.4)				
	LM-l^*^	LM-II^*^	FD^*^	BD^*^	CDT-0^*^	CDT-C^*^	FAS-F^*^	FAS-S^*^	**TMT-A**	**TMT-B**	Cards^*^	BNT^*^	**VOSP**	**Apraxis**
CNT	40.3 (8.4)	26.5 (7.9)	8.9 (2.7)	6.0 (2.0)	7.0 (0.0)	7.0 (0.0)	15.2 (3.9)	17.3 (3.7)	23.8 (0.9)	21.2 (3.3)	3.3 (1.0)	55.9 (3.9)	9.3 (2.9)	7.9 (0.2)
MCI	20.3 (11.3)	8.5 (8.7)	6.4 (1.5)	4.7 (1.1)	6.0 (1.6)	6.2 (1.7)	10.8 (3.2)	12.9(2.8)	22.6 (5.6)	18.2 (7.8)	2.3 (1.4)	51.3 (7.5)	8.5 (3.6)	7.2 (1.9)

*Left and right hippocampual volume (Left HV; Right HV), Age, Sex (F, female; M, male), Education, Minimental state examination (MMSE), Global dementia scale (GDS), Functional activity questionnaire (FAQ), Geriatric depression scale (GDS-15), Immediate and delayed memory test (LM-I, LM-II), Forward and backward digit span test (FD, BD), Clock drawing test, order and copy (CDT-O, CDT-C), phonetic and semantic verbal fluency test (FAS-F, FAS-S), Trail making test A and B (TMT-a, TMT-B), Rule shift cards (Cards), Visual Object and Space Perception Test (VOSP), and Ideomotor apraxis test (Apraxis)*.

* *Asterisk indicates significant difference between groups (p < 0.05). (1) GDS scores: CNT = 1, MCI = 3 (no statistics are computed), (2) FAQ between groups, p = 0.054, and (3) CDT-C between groups, p = 0.05*.

### Diagnostic criteria

All of the participants were rated with a variety of standardized diagnostic instruments that included the following: the Spanish version of the mini-mental state examination, MMSE (Lobo et al., 1979), the Geriatric Depression Scale (GDS-15; Yesavage et al., 1982), the Global Deterioration Scale (GDS; Reisberg et al., 1982), the Hachinski Ischemic Score (HIS; Rosen et al., 1980), the Functional assessment questionnaire (FAQ; Pfeffer et al., 1982), and the questionnaire for Instrumental Activities of Daily Living (IADL; Lawton and Brody, 1969).

**Figure 1:**
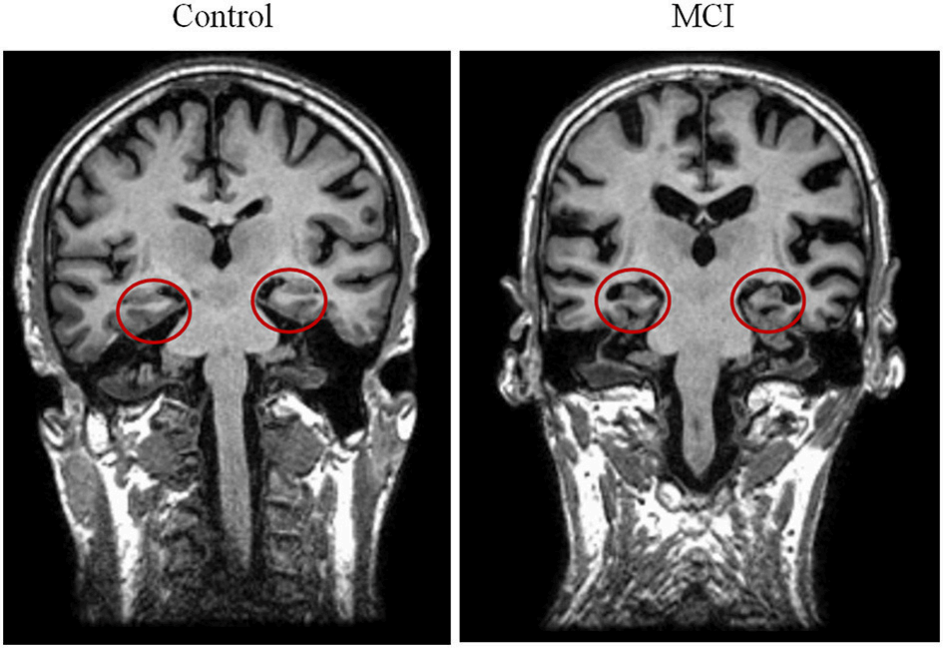
**Two illustrative T1 MRI images of one control (left) and one MCI (right) participant**. The red circles highlight the hippocampal volumes and evidence atrophy in the group of patients.

MCI diagnosis was established according to the National Institute on Aging–Alzheimer Association (NIA–AA) criteria (Albert et al., 2011): (1) self- or informant-reported cognitive complaint; (2) objective evidence of impairment in one or more cognitive domains; (3) preserved independence in functional abilities; and (4) not demented (McKhann et al., 2011). Besides meeting the core clinical criteria for MCI, subjects showed a positive biomarker reflecting neuronal injury (hippocampal volume reduction) which was measured by MRI (see Figure 1). So, the MCI group could be categorized as MCI attributable to AD-intermediate likelihood.

All subjects underwent an extensive neuropsychological assessment to evaluate their cognitive status in multiple areas with the following tests: clock drawing test (Agrell and Dehlin, 1998), direct and inverse digit span test [Wechsler Memory Scale (WMS-III); Wechsler, 1987], immediate and delayed recall (WMS-III; Wechsler, 1987), phonemic and semantic fluency (controlled oral word association test; Benton and Hamsher, 1989), ideomotor apraxis of Barcelona test (Peña-Casanova, 1990), rule shift cards (behavioral assessment of the dysexecutive syndrome; Norris and Tate, 2000), visual object and space perception test (VOSP; Warrington and James, 1991), Boston naming test (Kaplan et al., 1983), and trail-making tests A and B (Reitan, 1958).

According to their clinical and neuropsychological profile patients were diagnosed as amnesic-multidomain MCI (Petersen, 2004).

Other inclusion criteria were the absence of significant cerebral-vascular disease (i.e., modified Hachinski score ≤ 4) or depressive symptomatology (i.e., GDS-15 score ≤ 5). The participants were not using drugs that could affect cognitive performance (including cholinesterase inhibitors). All participants were free of significant medical, neurologic and/or psychiatric diseases other than MCI.

Prior to the study, all of the participants gave a written informed consent to participate in the investigation. The study was approved by the local ethics committee.

### Hippocampal volumes

Hippocampal volumes were measured as anatomical evidences of brain atrophy characteristic for MCI and AD (Albert et al., 2011; McKhann et al., 2011). For most of the subjects included in this paper (15 MCIs and 10 controls), a T1-weighted MRI was available, acquired in a GE Healthcare 1.5 Tesla magnetic resonance scanner, using a high-resolution antenna, and a homogenization pure filter (fast spoiled gradient echo sequence; repetition time, 11.2 ms; echo time, 4.2 ms; inversion time, 450 ms; flip angle, 12°; 1 mm slice thickness; 256 × 256 matrix; and field of view, 25 cm). These MRI images were processed with Freesurfer software (version 5.1.0) and its specialized tool for automated cortical and subcortical segmentation (Fischl et al., 2002) to obtain the volume of several brain areas, including hippocampus. Finally, volumes were normalized with respect to the individual intracranial volume (ICV) to account for differences in head volume over subjects.

### Stimuli

Trial-unique neutral, anonymous male and female faces across a large age range were used as stimuli in the current experiment. The hair and ears were removed digitally to avoid non-face-specific cues. The experiment was computerized through E-prime1.2 software (Psychology Software Tools, Inc.).

### Experimental paradigm

**Figure 2:**
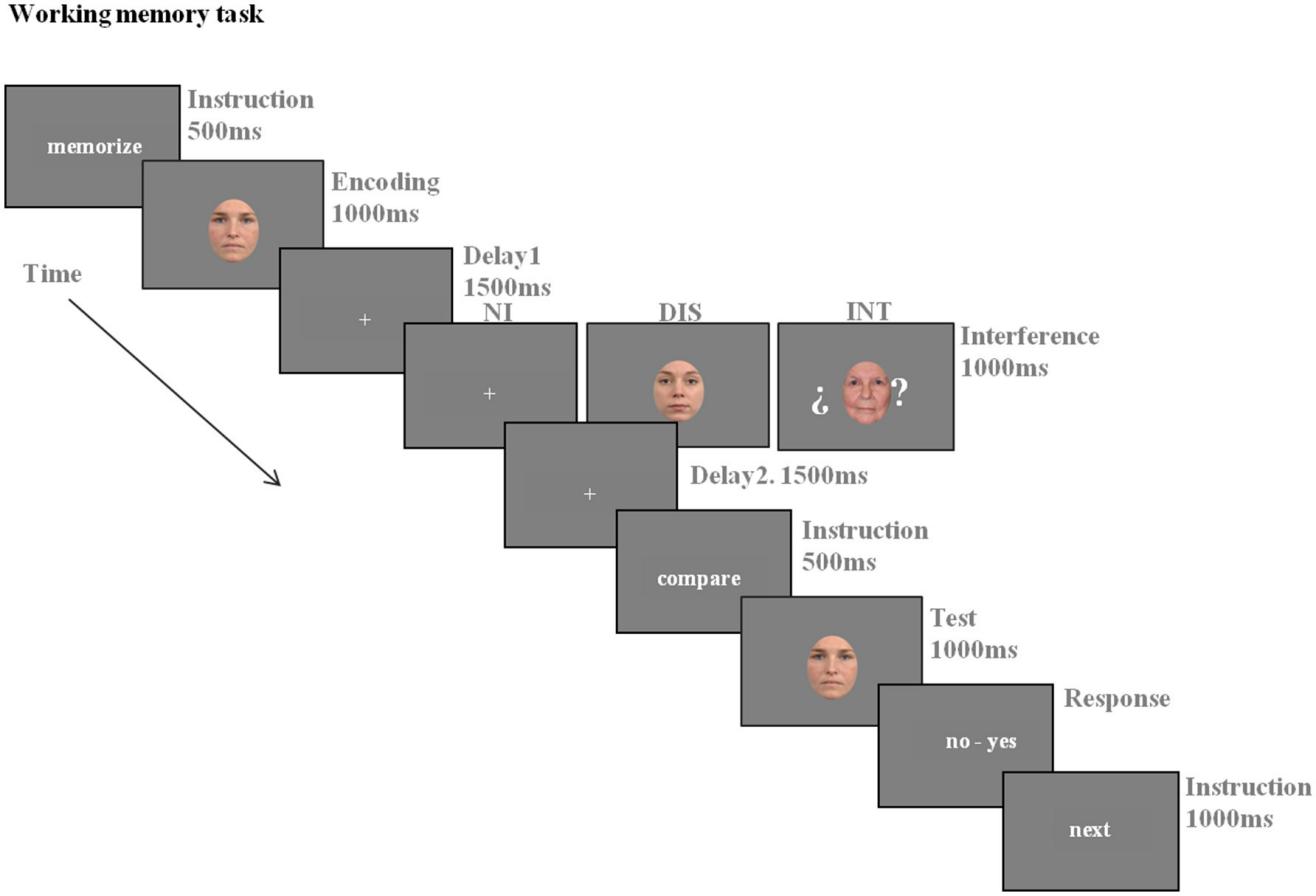
**The working memory task consisted of three conditions: Non-Interference (NI), Distraction (DIS), and Interruption (INT)**. All of the conditions were structured in three main phases: encoding, delay and recognition (See Experimental Paradigm section for details).

The participants performed a delayed match-to-sample task with three conditions: non-interference (NI), distraction (DIS), and interruption (INT; see Figure 2 for a representation of the paradigm). According to previous aging studies using very similar tasks (Solesio-Jofre et al., 2011; Clapp and Gazzaley, 2012) each condition was presented in a block. Each block consisted of 32 randomly presented trials resulting in a total of 96 trials per participant. The block presentation order was counterbalanced across subjects.

Before the experiment, participants were instructed about the task and also carried out a practice session to ensure an adequate understanding. All of the conditions consisted of three main phases: encoding, maintenance, and recognition. In the encoding phase, a face was displayed for a 1000 ms period and the participants were instructed to encode it. In the maintenance period, the participants were instructed to keep the encoded face in mind for a 4000 ms delay period. In the recognition phase, a single face was displayed for 1000 ms. Participants were instructed to make a match/non-match button press as rapidly as possible, without sacrificing accuracy, to indicate if the face was the same as the one presented in the encoding phase. To ensure that all of the participants got enough time to respond, a response slide (“no–yes”) was displayed after the recognition phase and maintained until the participant made the button press. This response slide was followed by the instruction “next” for 500 ms, which indicated the step to the next trial. Both the encoding and retrieval phases were preceded by the instructions “memorize” and “compare” for 500 ms each, respectively, to ensure adequate orientation within each phase of the task. The instruction regarding the maintenance phase varied between the conditions, depending on the presence or not of a distraction or interruption stimulus. In the NI condition, a fixation cross was displayed in the center of the screen for the 4000 ms of maintenance period, and the participants were instructed to keep the encoded face in mind during this period. In the DIS condition, a face stimulus was added as a distractor for 1000 ms after the first 1500 ms of the maintenance period. The participants were instructed to ignore the distractor while continuing to focus on the computer screen during the presentation of the distractor, and while maintaining the encoded face. The INT condition included a face stimulus as an interruption after the encoding phase, which was displayed for 1000 ms after the first 1500 ms of the maintenance period. The participants were instructed to make a button press if the interruption face was judged to be over 60 years of age. If not, they did not press any button. The interrupting face was presented between two question marks, indicating the additional requirement to process and respond to the stimulus. All participants responded to the interruption face with a minimum of 75% of correct responses.

### Statistical analysis

The neuropsychological scores were compared between healthy and MCI groups by means of independent sample *t*-tests.

The analysis of the WM task was based on correct responses for each condition and group. The statistical analysis of the accuracy and reaction time (RT) was performed by means of two-way repeated measures ANCOVA using a within-group factor of condition (NI, DIS, and INT), a between-group factor of diagnosis (controls and MCIs) and the FAQ scores as covariable (given a trend toward significance between the groups. See Table 1). The resulting *p*-values of pairwise comparisons were corrected using the *Bonferroni* procedure.

To further examine the differential impact of interference on WM performance (accuracy and RT), the scores regarding the DIS and INT conditions were normalized to the performance of the NI condition. Thus, the effect of distraction and interruption was calculated for each group using Z scores: *Effect of Interference = mean [(I – X)/SE]*, where *I* was the accuracy/reaction time during DIS or INT for each participant and *X* and *SD* were the mean and standard error values of each group for NI. Once the Z scores were calculated, independent sample *t*-tests were performed to examine potential differences between the groups.

## Results

### Neuropsychology

Analysis of the demographic and neuropsychological data revealed significant lower scores in the MCI group, compared with the control group, as indicated by asterisks in Table 1.

### Working memory

#### Accuracy

**Figure 3:**
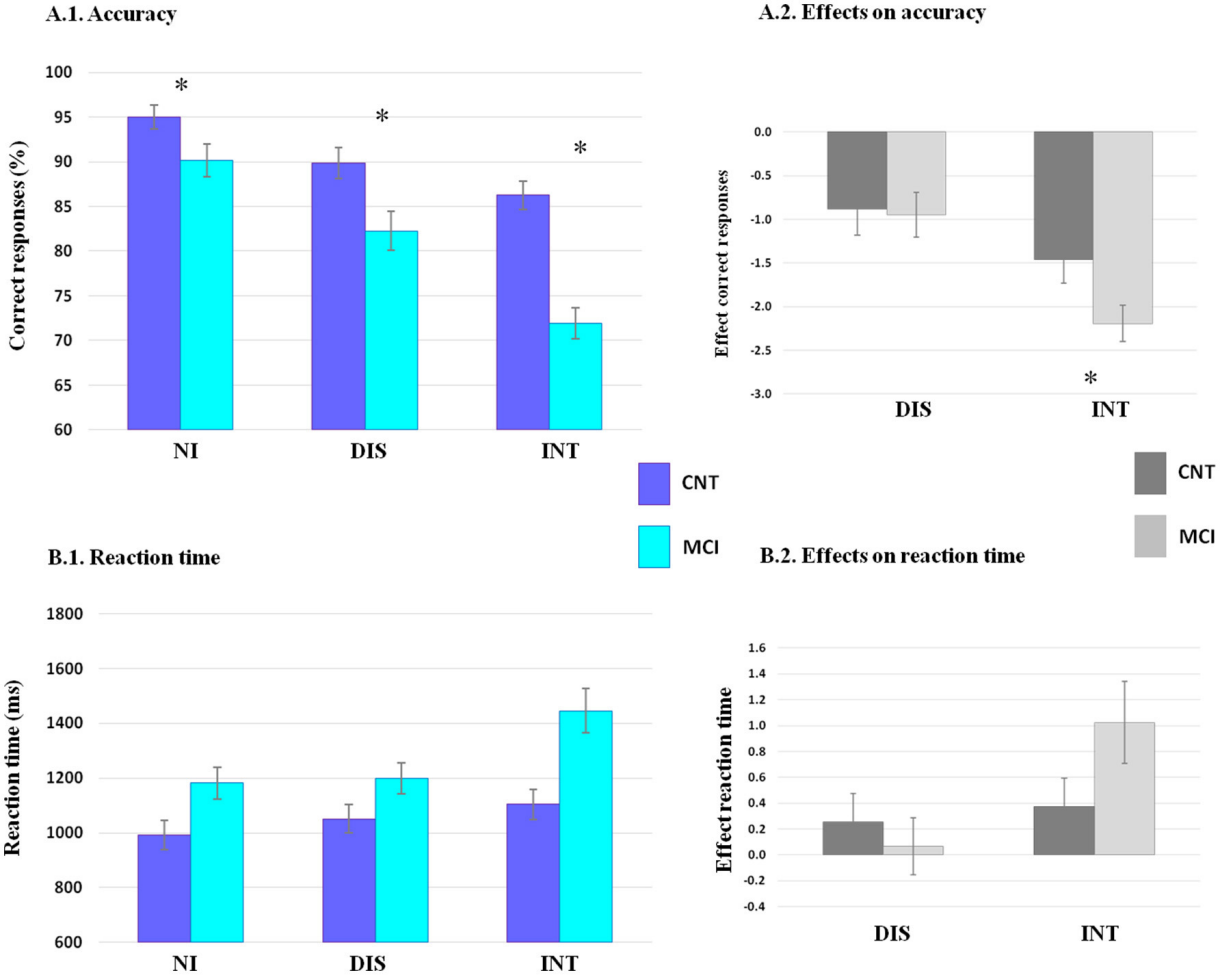
**Results for accuracy and reaction time (RT) are shown**. The percentage of correct responses **(A.1)** and RT in milliseconds **(B.1)** are shown for each condition (non-interference, NI, distraction, DIS, and interruption, INT) in each group (controls, CNT, dark blue, and MCIs in light blue). The effects of interference on accuracy **(A.2)** and RTs **(B.2)** are shown for distraction (DIS) and for interruption (INT) in each group (controls, CNT, in dark gray and MCIs in light gray). Error bars denote standard error of the mean. Statistical analysis of the accuracy revealed main effect of diagnosis and condition (*p* < 0.001), and a condition × group interaction (*p* < 0.005). The effect of interruption on accuracy was significantly greater in patients than with controls (*p* < 0.05). Reaction times showed main effect of diagnosis and condition (*p* < 0.001). ^*^indicates significant difference between groups (*p* < 0.05).

See Figure 3A for accuracy data. Mauchly's test indicated that the assumption of sphericity had not been violated [$\chi_{\left(2\right)}^{2}$ = 0.8, p = 0.67]. Analysis of the accuracy resulted in a main effect of diagnosis [*F*_(1, 33)_ = 16.19; *p* < 0.001; η^2^ = 0.33], with lower performance in MCIs than in controls. Also, a main effect of condition was revealed [*F*_(2, 66)_ = 30.04; *p* < 0.001; η^2^ = 0.54], such that all participants showed the greatest accuracy in the NI condition, reduction in accuracy in the DIS, and the lower accuracy during the INT condition. In addition, a significant two-way interaction of condition x diagnosis [*F*_(2, 66)_ = 5.5; *p* < 0.005; η^2^ = 0.14] was observed.

Within-group comparisons revealed a reduction in accuracy by distraction and interruption in the two groups (all *p* < 0.05). Furthermore, interruption elicited a greater impact than distraction in the group of patients (*p* < 0.001) but not in controls (*p* > 0.05). Comparisons between groups revealed lower performance in MCI patients across the three conditions (all *p* < 0.05).

#### Reaction time

See Figure 3B for reaction time data. Mauchly's test indicated that the assumption of sphericity had been violated [$\chi_{\left(2\right)}^{2}$ = 11.23, *p* = 0.004] and therefore, degrees of freedom were corrected using Greenhouse–Geisser estimates of sphericity (ε = 0.77). Analysis of the RTs resulted in a main effect of diagnosis [*F*_(1, 33)_ = 8.88; *p* < 0.01; η^2^ = 0.21], with slower RTs in MCIs than in controls; and a main effect of condition [*F*_(2, 66)_ = 7.4; *p* < 0.005; η^2^ = 0.18], where all participants showed the largest RTs in the INT condition, intermediate RTs in the DIS and the fastest RTs in the NI condition. Additionally, data revealed a trend toward a two-way interaction of condition × diagnosis [*F*_(2, 66)_=3.29 *p* = 0.057; η^2^ = 0.091].

#### Effect of interference on accuracy between groups

This analysis showed a disproportionate accuracy depletion by interruption in patients comparing with controls [*Effect-interruption*; *t*_(38)_ = 2.1, *p* < 0.05]. In contrast, no significant differences were observed between groups regarding distraction [*Effect-distraction*; *t*_(38)_ = 0.1, *p* > 0.1].

#### Effect of interference on reaction time between groups

This analysis revealed no significant differences in RT between groups neither by distraction [*Effect-distraction*; *t*_(38)_ = 0.5, *p* > 0.1], nor by interruption [*Effect-interruption*; *t*_(38)_ = 1.6, *p* > 0.1].

## Discussion

The current study explored the effect of two types of interference, distraction and interruption, during the performance of a visual WM task in MCI patients and healthy elder volunteers. Results showed that interference affected WM (Clapp et al., 2010) in both populations but in a different manner. While both MCI and control participants were similarly affected by distraction, MCI patients were disproportionately affected by interruption. These results show novel evidence for the negative impact of interference on WM in individuals with MCI.

The neuropathology of MCI has been largely observed in medial lobe structures, such as the hippocampus (Hyman et al., 1984; Tabert et al., 2006). In accordance, its clinical symptomatology has been most frequently described as an impaired ability to maintain and retrieve information from memory (Welsh et al., 1992; Petersen et al., 1999). Our results support this evidence by showing significantly reduced hippocampal volume (bilaterally), and reduced immediate and delayed recall of information in traditional episodic memory task (WMS-III) in patients when compared with controls.

In addition, our data are consistent with previous studies reporting difficulties to maintain information during short time periods in MCIs (Alescio-Lautier et al., 2007; Belleville et al., 2007; Kessels et al., 2010; Gagnon and Belleville, 2011). Nevertheless, the current experimental task tries to go one step beyond by evaluating how the WM retention ability in MCI is further affected by external stimuli. In agreement with the current results, both distraction and interruption have been associated with decrements in WM due to aging (Clapp and Gazzaley, 2012). However, the main results of the current study reveal, a WM vulnerability to distraction and interruption and a high susceptibility to the last in MCI patients. These results are in accordance with the neuropsychological profiles showing a dysexecutive profile in this group of amnesic-multidomain MCI individuals. Executive processing, including control of interference, relies on the prefrontal cortex (PFC) and on its interplay with posterior regions of the brain (Miller and Cohen, 2001). In this line, in addition to the involvement of the medial temporal lobe structures in the cognitive profile of patients with MCI (Petersen et al., 2006), affection of prefrontal regions are evident as well. Amyloid deposition, volume changes, altered activation, and connections of the PFC with other brain regions are some of the features of MCIs (Bell-McGinty et al., 2005; Maestú et al., 2008; Chao et al., 2009; Okello et al., 2009; Bajo et al., 2010). This evidence explains why impairments in executive processing, especially in inhibition, are often observed in the clinical profile of MCI (Wylie et al., 2007; Bélanger and Belleville, 2009; see Johns et al., 2012 for a review), and also in AD (Collette et al., 1999, 2009; Amieva et al., 2004; Belleville et al., 2007). Indeed, changes in the structure and function of the PFC in individuals with MCI (especially in multi-domain) have been specially related with alterations in executive control skills (Grambaite et al., 2011; Zheng et al., 2014). These evidences suggest that alterations in the PFC of our MCI individuals might underlie altered mechanisms to control interference and in consequence, lead to forgetting in WM.

In an attempt to explore the neural patterns of the control of distraction during WM in amnesic-multi-domain MCIs, Deiber et al. (2011) explored the electroencephalographic (EEG) activity of such a population while they performed a WM task. The experimental design included two faces and two letter stimuli presented in an interspersed manner. The stimuli had to be encoded or suppressed depending on the instructions before each trial. The results revealed reduced right central alpha frequency synchronization in response to distracting letters in the MCI group compared to the controls. Authors concluded that this hypo-synchronization reflected altered mechanisms of suppressing irrelevant stimuli. These results support the current data showing that WM depletion in MCI patients is exacerbated by an alteration to inhibit irrelevant stimuli.

Regarding interruption, our results show that even when both groups present larger latencies to perform the INT condition, the accuracy of the MCIs becomes disproportionately affected in this condition (confirmed by the *Effect on interruption*). These data evidence a special susceptibility of MCI individuals to maintain memoranda in the presence of stimuli that require additional processing resources. Previous research reveals difficulties in MCIs when attempt to perform two tasks simultaneously (Albert et al., 2001; Belleville et al., 2008b; Borkowska et al., 2009; Lonie et al., 2009; Sinai et al., 2010; Clément et al., 2012). Belleville et al. (2007) studied attentional control skills in MCI patients under three experimental tasks. The most significant results were found within a modified version of the Brown-Peterson procedure (Morris, 1986). In such a task, information is maintained over short delays while a second task (completing addition) is performed. The results showed that attention to the secondary task affects memory performance in MCI. Furthermore, memory accuracy was related to overall severity suggesting a gradual decline of memory and interference resolution in the continuum to AD. In a similar study, Alescio-Lautier et al. (2007) evaluated the memory recognition abilities of MCI and AD patients during delay periods that were interference-free or filled by a secondary task (interruption). Both groups of patients showed WM depletion in the presence of interference. In accordance with their results, the current data claim to an affection of WM when engaging and disengaging attention between interference and memoranda.

From the current data, a strong claim about the neural mechanisms that underlie WM affection by interruption is not straightforward. Nevertheless, evidence shows the involvement of the PFC in the top-down control of attention over posterior regions of the brain (Miller and Cohen, 2001). On the one hand, handling distraction during WM requires attentional and inhibitory control mechanisms which allow maintaining the relevant information and voluntarily inhibiting the irrelevant (Hedden and Park, 2001). On the other hand, handling interruption during WM requires, in addition, attention switching abilities which allow handling attention between the memoranda and the secondary task (Sakai et al., 2002a,b; Clapp et al., 2011). Altogether, it seems reasonable that the WM depletion of MCIs might be highlighted by difficulties in inhibition and in switching attention between stimuli, probably caused by alterations in the PFC and/or in the prefrontal-posterior network of the brain. Empirical evidence about the role of this brain network in WM abilities in MCIs should be addressed in future neurophysiological studies.

One limitation of the current study was that not all the hippocampal volumes were accessible. However, given that statistical significance was observed using 75% of the patients and 50% of the controls, similar results to the current ones will be expected when comparing the data from all the participants. A second limitation of the current investigation refers to the effect size of the condition × group interaction. Increasing the number of participants in future studies will confirm the present findings.

In sum, the current study indicates that difficulties in suppressing distraction and switching attention between memoranda and external stimuli play a fundamental role in WM performance in amnesic-multidomain MCI patients, and that these failures are modulated by the cognitive demands of interference. Thus, the disproportionate vulnerability of MCI patients to a high demanding interference condition points to its relevance when assessing WM ability in this clinical population. Given the high amount of relevant and irrelevant information in our daily living activities, future research should keep investigating the relation between the attentional control and WM mechanisms that contribute to forgetting in MCI patients.

## Author contributions

Design of the experiment: SA, JG, JF, FM, ML. Acquisition of data: SA Analysis of data SA, JG, DD, JP. Clinical evaluation of participants: AM, MD. Interpretation of data and Drafting of manuscript: SA, JG, DD, ML, FM.

### Conflict of interest statement

The authors declare that the research was conducted in the absence of any commercial or financial relationships that could be construed as a potential conflict of interest.
